# Evaluation of Galectin-3 in Dogs with Atrial Fibrillation

**DOI:** 10.3390/ani14172547

**Published:** 2024-09-02

**Authors:** Giulia Arcuri, Carlotta Valente, Giovanni Romito, Federico Bonsembiante, Chiara Mazzoldi, Barbara Contiero, Helen Poser, Carlo Guglielmini

**Affiliations:** 1 Department of Animal Medicine, Production and Health, University of Padua, 35020 Padua, Italy; giulia.arcuri@phd.unipd.it (G.A.); carlotta.valente@unipd.it (C.V.); federico.bonsembiante@unipd.it (F.B.); barbara.contiero@unipd.it (B.C.); helen.poser@unipd.it (H.P.); 2 Department of Veterinary Medical Sciences, University of Bologna, 40064 Bologna, Italy; giovanni.romito2@unibo.it (G.R.); chiara.mazzoldi2@unibo.it (C.M.)

**Keywords:** canine, biomarker, cardiac disease, echocardiography

## Abstract

**Simple Summary:**

Cardiac fibrosis is a common manifestation of heart disease that leads to the deterioration of cardiac function and the development of cardiac arrhythmias. Atrial fibrillation (AF) is particularly influenced by cardiac fibrosis, which is considered one of the primary factors in its development. Various diagnostic techniques can be employed to assess myocardial fibrosis, including cardiac imaging and the evaluation of circulating biomarkers. Among these biomarkers, galectin-3 (Gal-3) is notable for its involvement in inflammation and tissue fibrosis associated with cardiac disease. In humans, several studies have reported that an increased serum Gal-3 concentration is a risk factor for AF. In this study, we evaluated the serum concentration of Gal-3 in 26 dogs with AF associated with acquired cardiac diseases. A total of 17 cardiac healthy dogs and 30 dogs with cardiac disease but without AF served as controls. Our findings indicated no significant difference in Gal-3 concentrations between healthy dogs and dogs with cardiac disease, regardless of the presence of AF. Gal-3 showed a significant positive correlation with age. The results of this study suggest that Gal-3 does not have an important role for the development of AF in dogs, but it is associated with advanced age.

**Abstract:**

Galectin-3 (Gal-3) is a lectin associated with fibrosis and inflammation, and increased circulating concentrations are considered a risk factor for atrial fibrillation (AF) in humans. This retrospective study aimed to evaluate the serum concentration of Gal-3 in dogs with cardiac disease, both with and without AF. Dogs with AF associated with acquired heart diseases were selected, while cardiac healthy dogs and dogs with heart diseases but without AF served as controls. We statistically compared the serum concentration of Gal-3, which was assessed using a commercial canine-specific ELISA kit, among healthy dogs and dogs with heart disease with and without AF. Additionally, associations between Gal-3 and clinical and echocardiographic variables were evaluated. A total of 73 dogs were included, of which 17/73 (23.3%) were cardiac healthy and 56/73 (76.7%) had heart disease, with 26/56 (46.4%) having AF. No significant difference in Gal-3 concentration was found between cardiac healthy dogs (3.90 ± 1.65 ng/mL) and dogs with heart disease, either with or without AF (3.37 ± 1.04 ng/mL, *p* = 0.436 and 4.68 ± 1.80 ng/mL, *p* = 0.332, respectively). Gal-3 showed a significant positive correlation with age (r = 0.47, *p* < 0.001) and a negative correlation with body weight (r = −0.45, *p* < 0.001). The results of this study suggest that Gal-3 does not have an important role in the development of AF in dogs, but it is associated with advanced age.

## 1. Introduction

Galectin-3 (Gal-3) is a beta-galactoside-binding protein belonging to the lectin family that is released by activated macrophages. It plays a vital role in many physiological cellular functions, including cellular growth, differentiation, proliferation, apoptosis, cellular adhesion, and tissue repair [1].

In human medicine, increased Gal-3 levels have been reported to be associated with a variety of disorders, including congestive heart failure (CHF), renal failure, diabetes mellitus, and cancer [2,3,4,5]. Its involvement in the pathogenesis of cardiovascular diseases has been extensively studied, particularly with reference to its role in inflammatory processes and tissue fibrosis. An increase in Gal-3 concentration stimulates the release of several mediators that promote cardiac fibroblast proliferation, collagen synthesis and deposition, and ventricular dysfunction [5,6,7,8]. Consequently, Gal-3 is considered a biomarker that is predictive of cardiac remodeling and adverse cardiac events, including the risk of the onset of CHF and myocardial fibrosis [5,9,10,11,12].

The inflammation and fibrosis of the myocardium are particularly important in the etiology of atrial fibrillation (AF). The pathophysiology of AF is complex and involves, among other factors, atrial pro-inflammatory responses that lead to electrical and structural remodeling associated with myocardial fibrosis. This process creates a pro-arrhythmic substrate that promotes the onset of AF [13,14,15]. Due to these mechanisms, several studies have investigated the possible relationship between the serum concentration of Gal-3 and the risk of developing AF in humans, revealing a correlation between increased Gal-3 levels and an increased risk of AF [16,17,18,19].

In veterinary medicine, a few recent studies have evaluated the role of Gal-3 in dogs and cats with cardiac and noncardiac diseases, such as endocrine, dermatologic, or neoplastic disorders [20,21,22,23]. Specifically, some studies have demonstrated an increased Gal-3 concentration in dogs with congenital or acquired heart disease, including myxomatous mitral valve disease (MMVD) and dilated cardiomyopathy (DCM) [20,24,25,26,27,28]. As in humans, AF is the most common supraventricular arrhythmia in dogs [29,30,31,32] and typically occurs secondarily to cardiac diseases associated with left atrial enlargement [33,34]. Additionally, AF is usually long-standing, persistent, or permanent in dogs and its development is associated with a worse prognosis in dogs with MMVD and DCM [35,36,37]. Previous studies reported on clinical and echocardiographic parameters associated with the development of secondary AF in dogs [33,34,35,36,37,38,39,40,41], whereas the evaluation of laboratory parameters associated with atrial remodeling and fibrosis is lacking in these animals. Thus, investigating the correlation between the serum Gal-3 concentration and AF can be useful in the clinical evaluation of dogs with heart disease. To the authors’ knowledge, no studies have yet investigated the relationship between Gal-3 and AF in dogs.

Therefore, the aim of this study was to evaluate the serum concentration of Gal-3 in dogs with cardiac disease, with or without AF. We hypothesized that the presence of AF would be associated with an increased serum concentration of Gal-3.

## 2. Materials and Methods

### 2.1. Animals

In this retrospective study, clinical data of dogs visited from July 2017 to October 2023 at the Cardiologic Units of the Veterinary Teaching Hospital (VTH) of the University of Padua and Bologna were reviewed. All consecutive cases of dogs with MMVD or DCM associated with AF were selected.

The diagnosis and classification of heart disease were performed based on previously described clinical and echocardiographic criteria [42,43,44,45,46,47]. The presence or absence of AF was based on a 6-/12-lead surface ECG with at least a 3 min duration. Specifically, the diagnosis of AF was based on the combined presence of the following findings: the lack of recognizable P waves and an irregular cardiac rhythm with narrow QRS complexes [48,49].

Cardiac healthy dogs (i.e., dogs without heart disease) and dogs with heart disease but without AF were included in the study as control groups. These control animals were selected from the database of the same VTHs during the same time period. Specifically, cardiac healthy dogs had normal results of a thorough cardiac evaluation, including a echocardiographic examination, unremarkable CBC, and biochemical profile findings. Additionally, these dogs were matched by age and body weight with dogs with MMVD (i.e., the largest group of dogs with AF). Control dogs with heart disease but without AF were chosen among animals with the same type and stage of cardiac disease as those with AF, and were cross-matched with them by sex, age, and bodyweight. The presence of concomitant noncardiac disease was not an exclusion criterion for all dogs included in the study, but this information was noted.

Each dog included in this study underwent blood sampling for routine CBC and biochemical profile. Serum samples were kept at room temperature for 15 min to allow for stable clot formation and then were centrifuged for 10 min. An aliquot of serum was then harvested and frozen at −80 °C until the batch analysis for the evaluation of the Gal-3 concentration.

### 2.2. Echocardiographic Examination

At each VTH, an experienced operator (G.R., H.P., and C.G.) performed the echocardiographic examination using echocardiographic units (iE33 and CX50 ultrasound systems, Philips Healthcare, Eindhoven, The Netherlands) equipped with dedicated multifrequency phased array transducers (S5-1 and S3-8 MHz) and continuous ECG tracing.

The measurements of left ventricular diastolic diameter (LVDD) and left ventricular systolic diameter (LVSD) were obtained from M-mode short-axis echocardiographic images at the level of the chordae tendineae. Left ventricular diastolic and systolic measurements were then transformed using the described allometric scaling system to obtain normalized measurements for body weight (LVDDn and LVSDn, respectively) [50]. Fractional shortening (FS) was then calculated using the standard formula. Left atrial diameter (LA) and aortic root diameter (Ao) were measured at early diastole from 2D echocardiographic short-axis images obtained at the level of the heart base, and the LA:Ao ratio was then calculated [51]. The trans-mitral blood flow was examined using the pulsed-wave Doppler technique from the left apical four-chamber view by positioning the sample volume at the tip of the mitral valve leaflets, and the peak velocity of the early diastolic wave (E Mitral) was obtained. All measurements were replicated on 3 or 5 consecutive beats, in dogs without or with AF, respectively, and the mean values were calculated.

### 2.3. Measurement of Serum Galectin-3 Concentration

The galectin-3 concentration was measured in canine serum samples using a commercially available ELISA kit (RayBio Canine Galectin-3 ELISA kit, RayBiotech, Norcross, GA, USA) with a detection range of 2–500 pg/mL (0.002–0.5 ng/mL). According to a previous study [28], serum samples were diluted 1:30 with the diluent included in the assay prior to analysis. This dilution ensured that all samples fell within the detectable range of the assay, and no additional dilutions were necessary. Measurements were performed following the manufacturer’s assay procedure, with all samples analyzed in duplicate. For the data analysis, the mean of the two measurements was utilized. Optical density was measured using a multimode microplate reader (Victor X4, PerkinElmer, Waltham, MA, USA), at a wavelength of 450 nm. The concentration of Gal-3 was obtained using the calibration curve, which allows for the conversion of optical density into concentration values. The calibration curve consists of 7 points at decreasing concentrations starting from a standard solution of 500 pg/mL (500 pg/mL; 200 pg/mL; 80 pg/mL; 32 pg/mL; 12.8 pg/mL; 5.12 pg/mL; 2.05 pg/mL) plus the blank point (0 pg/mL). Once the optical density was converted into the concentration of Gal-3, the obtained values were multiplied by the dilution factor to obtain the actual value of Gal-3. The concentration of Gal-3 was expressed in ng/mL instead of pg/mL to avoid excessively large numbers and to make the text easier to interpret.

### 2.4. Statistical Analysis

Data were analyzed using commercial software (SAS 9.4, SAS Institute Inc., Cary, NC, USA). A sample size calculation was performed to determine the number of dogs to be included in the study. Based on results from previous studies that measured Gal-3 concentrations, we calculated the sample size to achieve a power of 0.8 and a significance level (α) of 0.05 [24,27]. These calculations indicated that groups ranging from 8 to 17 dogs would be sufficient to detect a difference in Gal-3 concentrations.

Demographic and clinical characteristics included breed, sex, age, bodyweight, presence of heart disease, and corresponding severity; these characteristics were evaluated according to the American College of Veterinary Internal Medicine (ACVIM) classification [43]. The animals received ongoing treatment at the time of examination; the presence of other concurrent diseases and the presence or absence of AF were determined. The following continuous echocardiographic variables were considered: LA, Ao, LA:Ao, LVDDn, LVSDn, FS, and E Mitral. Normality of data was assessed using the Shapiro–Wilk test. Comparisons were made between healthy dogs and dogs with heart disease, with and without AF, and between cardiac healthy dogs and dogs with DCM or MMVD. Additionally, in dogs with heart disease, comparisons were made between those with compensated and decompensated heart disease (ACVIM classes B1 + B2 and C + D, respectively [43,45]).

Normally distributed data were reported as means and standard deviation and were compared using one-way ANOVA. Non-normally distributed data were reported as median and range (minimum-maximum) and were compared using the Kruskal–Wallis test. Fisher’s exact test was used to compare categorical variables. Post hoc pairwise comparisons were performed using Bonferroni’s correction. Associations between variables were evaluated using Spearman’s rank correlation coefficient (r). For all analyses, a *p* value < 0.05 was considered significant, except for Bonferroni’s correction, where *p* ≤ 0.017 was considered significant.

## 3. Results

### 3.1. Study Population and Echocardiographic Parameters

A total of seventy-three dogs were included in this study, comprising twenty-six (37%) females (one spayed and twenty-six intact) and forty-six (63%) males (two castrated and forty-four intact). The mean age was 9.9 ± 2.98 years, and the median bodyweight was 24.2 kg (range 2.2–120 kg). Most dogs were purebred (56 dogs, 76.7%). The most frequently represented breed was the Doberman Pinscher (six dogs, 8.2%), followed by the Miniature Pinscher and Jack Russel Terrier (four dogs each, 5.5%), and by the American Staffordshire Terrier, Cavalier King Charles Spaniel, and Dogue de Bordeaux (three dogs each, 4.1%). Other breeds were represented by one or two dogs each.

Seventeen (23.3%) dogs were cardiac healthy, while fifty-six (76.7%) had cardiac disease, including sixteen (28.6%), and forty (71.4%) dogs with DCM, and MMVD, respectively. Among dogs with cardiac disease, 26 (46.4%) had AF, while 30 (53.6%) maintained a sinus rhythm. Of those with AF, eight dogs (30.8%) had DCM, and eighteen dogs (69.2%) had MMVD.

Twenty-two dogs (30.1%) had concurrent noncardiac diseases, including neoplastic (five dogs, 6.8%), dermatological (five dogs, 6.8%), neurological (four dogs, 5.5%), gastrointestinal (four dogs, 5.5%), endocrine (two dogs, 2.7%), orthopedic (two dogs, 2.7%), and other various (three dogs, 4.1%) diseases. Specifically, neoplastic diseases included chronic leukemia, intracranial mass, lung, intestinal, and hepatoid gland neoplasia (one dog each). Among dermatological diseases, two dogs each had otitis and food allergies, and one dog had atopic dermatitis. Neurological disorders included herniated intervertebral discs (two dogs), and Wobbler syndrome and Chiari-like syndrome (one dog each). In the gastrointestinal group, two dogs had food-responsive enteropathy and one dog each had an esophageal foreign body and immune-mediated enteropathy. Endocrine disease included hypoadrenocorticism, hyperadrenocorticism, and hypothyroidism (one dog each), while orthopedic disorders included joint pain and hip–elbow dysplasia (one dog each). Finally, three dogs had chronic kidney disease, pyorrhea, and peritoneal hernia (one dog each). At the time of the study enrollment, 49 (67.1%) dogs were receiving cardiac treatment (CT), noncardiac treatment (OT), or both. Among those receiving CT, thirty-one (73.8%) dogs were receiving diuretics (furosemide or torasemide), thirty-seven (88%) were receiving pimobendan, twenty-six (62%) were receiving ACE inhibitors (benazepril or enalapril), seventeen (40.5%) were receiving spironolactone, and eight were receiving (19%) digoxin.

Table 1 presents a comparison of clinical and echocardiographic variables between cardiac healthy dogs and dogs with cardiac disease with or without AF. Age and the echocardiographic variables LVDDn, LVSDn, FS, and E mitral were normally distributed, whereas bodyweight and the echocardiographic variables LA, Ao, and LA:Ao were not normally distributed. Dogs with AF were heavier (*p* = 0.003), predominantly in ACVIM stage C or D (*p* < 0.001), and had higher LA and E mitral values compared to healthy dogs or dogs with cardiac disease but without AF (*p* < 0.001 for both comparisons). There were no significant differences found regarding breed (*p* = 0.806) and mean age (*p* = 0.107) among these groups. Among dogs with cardiac disease, there was no significant difference regarding the presence of concurrent disease (*p* = 0.224) and type of treatment at the time of admission (*p* = 0.905 for CT and *p* = 0.552 for CT + OT) between those with or without AF.

Table 2 presents a comparison of clinical and echocardiographic variables between cardiac healthy dogs and dogs with different types of cardiac diseases. Dogs with DCM were predominantly purebred compared to those with MMVD and cardiac healthy dogs (*p* = 0.034). Males were more prevalent in dogs with DCM (*p* = 0.016). Dogs with MMVD were older, whereas dogs with DCM were heavier (*p* < 0.001 for both comparisons). A significant difference was found regarding ACVIM stages between dogs with MMVD and those with DCM (*p* = 0.017). Not surprisingly, dogs with cardiac disease had a higher LA value and LA:Ao ratio, normalized left ventricular diameters, and E mitral values compared to cardiac healthy dogs (*p* < 0.001 for all comparisons). Additionally, dogs with DCM had reduced FS, whereas dogs with MMVD had increased FS (*p* < 0.001). No significant differences were found regarding concurrent disease (*p* = 0.073) among the three different groups of dogs and treatments administered at the time of admission between dogs with DCM and MMVD (*p* = 0.999 for CT and *p* = 0.999 for CT + OT).

### 3.2. Serum Galectin-3 Concentration

**Figure 1:**
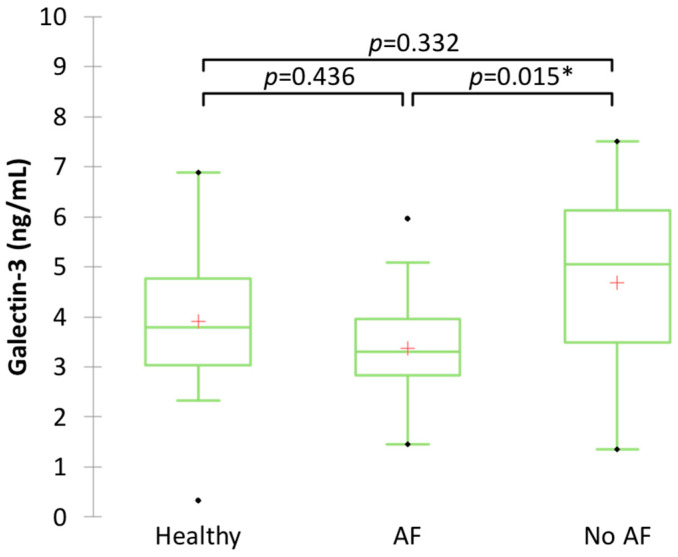
Boxplot showing serum Gal-3 concentration in clinically healthy dogs and dogs with heart disease with or without atrial fibrillation (AF). * Significant difference (*p* ≤ 0.017) between groups.

The mean serum Gal-3 concentration in dogs with cardiac disease without AF was 4.68 ± 1.80 ng/mL, which was significantly higher compared to that of dogs with cardiac disease with AF (3.37 ± 1.04 ng/mL, *p* = 0.015) (Figure 1). There was no significant difference in Gal-3 concentration between healthy dogs (3.90 ± 1.65 ng/mL) and dogs with cardiac disease with AF or without AF (*p* = 0.436 and *p* = 0.332, respectively).

**Figure 2:**
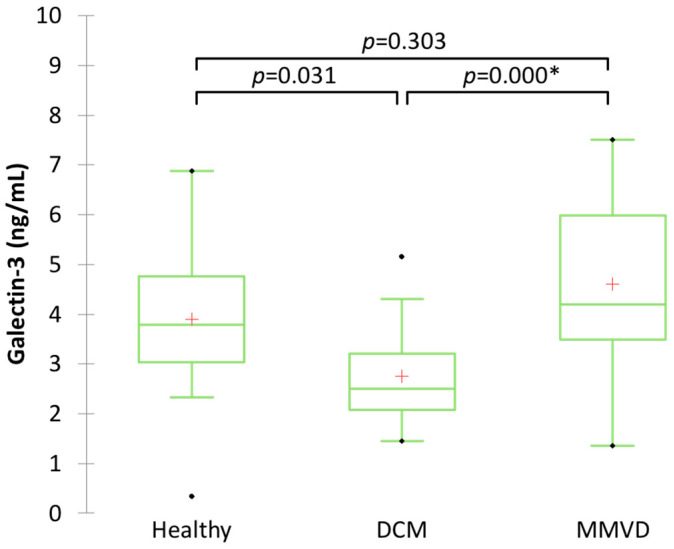
Boxplot showing serum Gal-3 concentration in clinically healthy dogs, dogs with dilated cardiomyopathy (DCM), and dogs with myxomatous mitral valve disease (MMVD). * Significant difference (*p* ≤ 0.017) between groups.

Among dogs with heart disease, animals with MMVD had a higher mean serum concentration of Gal-3 (4.61 ± 1.52 ng/mL) compared to that of dogs with DCM (2.75 ± 1.01 ng/mL, *p* = 0.000) (Figure 2). Additionally, there was no significant difference in the mean Gal-3 concentration between cardiac healthy dogs and dogs with heart disease (*p* = 0.031 for DCM, and *p* = 0.303 for MMVD).

In dogs with MMVD, those without AF had a higher mean serum concentration of Gal-3 compared to those with AF (5.35 ± 0.27 ng/mL and 3.69 ± 0.30 ng/mL, respectively; *p* < 0.01). Conversely, the mean serum concentration of Gal-3 in dogs with DCM was not significantly different between those with or without AF (2.65 ± 0.37 ng/mL and 2.84 ± 0.37 ng/mL, respectively; *p* = 0.716). Additionally, no significant difference in the mean serum concentration of Gal-3 was found between dogs with compensated heart disease (ACVIM stage B1–B2) and those with decompensated heart disease (ACVIM stages C–D) (4.13 ng/mL, 1.35–7.5 ng/mL and 3.78 ng/mL, 1.81–7.13 ng/mL, respectively, *p* = 0.595).

Table 3 shows the correlations between the serum concentration of Gal-3 and clinical and echocardiographic variables. Specifically, a significant positive correlation was found between Gal-3 and age (r = 0.47, *p* < 0.001) as well as fractional shortening (r = 0.43, *p* < 0.001). Additionally, there was a significant negative correlation between Gal-3 and body weight (r = −0.45, *p* < 0.001) and aortic root diameter (r = −0.38, *p* = 0.001).

## 4. Discussion

The main findings of this study were that the serum concentration of Gal-3 does not increase in dogs with secondary AF compared to those with cardiac disease maintaining a sinus rhythm. Among canine cardiac diseases, MMVD is associated with a higher Gal-3 concentration, and advanced age and low body weight are additionally correlated with an increased level of this biomarker in the dog.

The characteristics of dogs with AF secondary to cardiac disease in this study are consistent with those reported in previous studies [33,38,39,40]. Animals with AF were heavier and had higher LA and E mitral measurements than their counterparts maintaining a sinus rhythm. Atrial structural, electrical, ionic, and functional remodeling are the main cardiac modifications underlying the development of AF, both in humans and dogs [52,53,54,55]. Left atrial structural remodeling refers to adaptive or maladaptive changes in the cardiac architecture that occur at both macro and microscopic levels [53]. Specifically, atrial enlargement and fibrosis are the most important macroscopic and microscopic changes occurring during atrial remodeling in people with AF [53].

Few studies have investigated the atrial microscopic changes that occur in dogs with MMVD or DCM, the main ones being interstitial fibrosis, myocardial fat replacement, and immune cell infiltration [56,57]. Even fewer studies have evaluated microscopic changes at the atrial level in dogs with AF [56,58]. Beyond pathological studies, the clinical evaluation of atrial changes leading to or associated with AF is challenging. Echocardiography, including speckle-tracking echocardiography (STE), can be useful for identifying left atrial remodeling and dysfunction [41,59,60], but is not suitable to unveil atrial microscopic changes. Advanced imaging techniques, such as cardiac magnetic resonance imaging and computed tomography, allow for the accurate assessment of myocardial fibrosis [61,62,63], but are not routinely employed in the canine clinical setting.

Therefore, increasing attention has been paid to circulating molecules, such as Gal-3, as potential biomarkers of cardiac remodeling and fibrosis in recent years. Despite these premises, an increased Gal-3 concentration was not associated with the development of AF in this study. Indeed, dogs with heart disease and maintaining a sinus rhythm had a higher median serum concentration compared to those with AF. In humans, AF often occurs in elderly patients without recognizable cardiac disease (i.e., primary or lone AF) [13,64]. In these patients, cardiac fibrosis in the absence of any discernible heart disease is likely the major inciting mechanism for the development of the arrhythmia [65,66], leading to an increased serum concentration of Gal-3. Furthermore, cardiac fibrosis is proportional to the amount of myocardial tissue involved, which is intrinsically higher in the ventricles compared to the atria and, consequently, the concentration of fibrosis-related serum biomarkers, such as Gal-3, also follows this proportionality. Therefore, we hypothesize that atrial fibrosis was likely not sufficient to result in the increased serum concentration of Gal-3 in dogs of the present study with secondary AF.

Regarding the evaluation of Gal-3 according to different cardiac diseases, dogs with MMVD had a significantly increased Gal-3 concentration compared to dogs with DCM, regardless of the presence of AF, but not compared to cardiac healthy dogs. Cardiac fibrosis is a pathological feature of MMVD, especially at the level of papillary muscles and chordae tendineae [67,68]. Furthermore, one study reported evidence of fibrosis in the left atrium of dogs with MMVD, although a histopathological evaluation of the ventricles was not performed in the same animals [57]. Conversely, the main histologic features of canine DCM, the second most represented cardiac disease in dogs of this study, include the “fatty infiltration-degenerative” type, characterized by myofibril degeneration, vacuolization, and adipocyte clusters, and the “attenuated wavy fiber” type, characterized by atrophic myocardiocytes with a wavy appearance [69]. These different pathological features of the two most common canine-acquired cardiac diseases explain the observed increase of Gal-3 concentration in dogs with MMVD.

Previous studies have found a significantly increased Gal-3 concentration in dogs with MMVD compared to healthy animals [24,25,26], but in all these studies, the control group was composed of dogs that were significantly younger than those with MMVD. Conversely, in the present study, cardiac healthy dogs were cross-matched by age with those with MMVD, and both groups had a mean age greater than 10 years, whereas dogs with DCM were younger (mean age of 7 years). Furthermore, we found a significant positive correlation between Gal-3 concentration and advanced age. These findings provide evidence of the correlation between Gal-3 and aging in dogs, as already reported in humans [70,71]. At the cardiac level, an hallmark of aging is progressive ventricular remodeling characterized by myocardial hypertrophy, interstitial fibrosis, and ultimately ventricular dysfunction [72], although the underlying pathophysiological mechanisms are complex and not completely understood [73,74].

Another finding in this study was the negative correlation between Gal-3 and both body weight and aortic root diameter. These results contrast to those reported in humans, where Gal-3 has been found to be positively correlated with body mass index in patients with heart disease [75]. Older small-sized dogs, with or without MMVD, had higher levels of Gal-3 in this study, likely explaining the observed negative correlation between this molecule and body weight.

Because of its retrospective design, this study has several limitations. First, Gal-3 is not a specific cardiac biomarker, and increased circulating concentrations have been reported in other noncardiac diseases, such as diabetes mellitus, kidney disease, and cancer in humans [2,3,4]. Similarly, some studies have shown a role for this biomarker in dogs with chronic dermatological, endocrine, and neoplastic disorders [20,22]. In this study, some dogs presented with concurrent noncardiac diseases, including neoplastic, orthopedic, neurological, endocrine, and dermatological disorders. These comorbidities may have affected our results, but the influence of some of them on the circulating Gal-3 levels has not been previously reported. Moreover, no difference was found in the prevalence of these comorbidities either between dogs with or without AF or between dogs with DCM or MMVD. Second, the circulating levels of Gal-3 were not analyzed in comparison with histologically proven myocardial fibrosis or imaging techniques other than standard echocardiography, which is poorly sensitive for myocardial fibrosis. In humans, an accurate assessment of myocardial fibrosis can be obtained using cardiac magnetic resonance imaging, cardiac computer tomography, or STE [61,62,63,76]. However, these diagnostic tools are not routinely available and performed in dogs with cardiac disease. Third, dogs of different breeds, sizes, and cardiac diseases were included in the study and these factors could have influenced our results. Particularly, the positive and negative correlation observed between the Gal-3 concentration and age and bodyweight, respectively, should be considered in light of the well-known negative correlation between size and longevity in dogs [77,78]. Another possible limitation concerns the long storage time of some serum samples before the Gal-3 evaluation. No studies evaluated the stability of canine Gal-3 levels for long periods; however, a study demonstrated that Gal-3 levels are stable for at least one year and with two freeze–thaw cycles in humans [79]. In the present study, the samples had never been thawed before the Gal-3 measurement. Finally, different analytical ELISA kits have been used for the evaluation of circulating Gal-3 levels in dogs, and values obtained using different kits are not equivalent [24,25,28]. Therefore, it is important to note that a comparison of the absolute or reference values of circulating Gal-3 levels measured with different kits are not interchangeable.

## 5. Conclusions

In conclusion, Gal-3 does not have an important role in the development of AF secondary to acquired cardiac diseases in dogs. In our study population, advanced age and MMVD were associated with increased circulating Gal-3 levels, likely reflecting cardiac remodeling secondary to these conditions. Future prospective studies with a more homogeneous population of dogs are warranted to understand the relationship between circulating Gal-3 levels and canine AF as well as to elucidate the potential prognostic role of this biomarker in dogs with cardiac disease.

## Figures and Tables

**Table 1:** Clinical and echocardiographic data obtained from 73 dogs divided into three groups: cardiac healthy dogs and dogs with heart disease with or without atrial fibrillation (AF).

Variable	Category	Healthy Group (N = 17)	AF Group (N = 26)	No AF Group (N = 30)	Overall *p*-Value
Breed	Purebred, N (%)	13/17 (76%)	21/26 (81%)	22/30 (73%)	0.806
Sex	Male/Female	6/11 ^b^	19/7 ^a^	21/9 ^ab^	0.025
Age (years)	Mean ± SD	10 ± 3	9 ± 3	11 ± 3	0.107
Body weight (kg)	Median (min–max)	13.7 (2.2–48.5) ^b^	32.1 (4–120) ^a^	11.05 (4.8–48.2) ^b^	0.003
Concurrent disease	Yes, N (%)	9 (53%) ^a^	4 (15%) ^b^	9 (30%) ^ab^	0.042
ACVIM stages	C + D, N (%)	-	24 (92%)	12 (40%)	<0.001
Treatment ad admission	CT, N (%)	-	16 (62%)	20 (67%)	0.905
	CT + OT, N (%)	-	5 (19%)	3 (10%)	0.552
	NT, N (%)	-	5 (19%)	7 (23%)	0.963
LA (cm)	Median (min–max)	2.3 (1.4–3.7) ^c^	5.3 (3.3–8.5) ^a^	4.0 (2.8–6.7) ^b^	<0.001
Ao (cm)	Median (min–max)	1.7 (1.0–2.7) ^b^	2.5 (1.1–3.1) ^a^	1.7 (1.2–3.2) ^b^	0.001
LA:Ao	Median (min–max)	1.4 (1.2–1.9) ^b^	2.2 (1.7–4.2) ^a^	2.1 (1.5–3.2) ^a^	<0.001
LVDDn	Mean ± SD	1.38 ± 0.10 ^b^	2.01 ± 0.30 ^a^	2.08 ± 0.26 ^a^	<0.001
LVSDn	Mean ± SD	0.86 ± 0.12 ^b^	1.26 ± 0.34 ^a^	1.25 ± 0.31 ^a^	<0.001
FS (%)	Mean ± SD	34.9 ± 6.6	30.4 ± 14.8	36.1 ± 15.9	0.301
E Mitral (m/s)	Mean ± SD	0.67 ± 0.11 ^c^	1.41 ± 0.39 ^a^	1.15 ± 0.32 ^b^	<0.001

Data are expressed as mean ± standard deviation (SD) or median (range). Different superscript letters along rows mean significant different values (*p* ≤ 0.017); Abbreviations: N, number of dogs; ACVIM, American College of Veterinary Internal Medicine; CT, cardiac treatment; OT, other treatment, NT, no treatment; LA, left atrial diameter; Ao, aortic diameter; LA:Ao, left atrial diameter to aortic diameter ratio; LVDDn, left ventricular diastolic diameter normalized to body weight; LVSDn, left ventricular systolic diameter normalized to body weight; FS, fractional shortening; E mitral, mitral valve maximal E wave velocity.

**Table 2:** Clinical and echocardiographic data obtained from 73 dogs divided according to presence and type of heart disease.

Variable	Category	Healthy Group (N = 17)	DCM Group (N = 16)	MMVD Group (N = 40)	Overall *p*-Value
Breed	Purebred, N (%)	13/17 (76%) ^ab^	16/16 (100%) ^a^	27/40 (68%) ^b^	0.034
Sex	Male/Female	6/11 ^b^	13/3 ^a^	27/13 ^ab^	0.016
Age (years)	Mean ± SD	10 ± 3 ^a^	7 ± 2 ^b^	11 ± 3 ^a^	<0.001
Body weight (kg)	Median (min–max)	13.7 (2.2–48.5) ^b^	42 (32.6–120) ^a^	11 (4–72) ^b^	<0.001
Concurrent disease	Yes, N (%)	9 (53%)	4 (25%)	9 (23%)	0.073
ACVIM stages	C + D, N (%)	-	6 (38%) ^b^	30 (75%) ^a^	0.017
Treatment ad admission	CT	-	10 (63%)	26 (65%)	0.999
	CT + OT	-	2 (13%)	6 (15%)	0.999
	NT	-	4 (25%)	8 (20%)	0.960
LA (cm)	Median (min–max)	2.4 (1.4–4.2) ^b^	4.9 (3.7–6.6) ^a^	4.4 (2.8–8.5) ^a^	<0.001
Ao (cm)	Median (min–max)	1.7 (1.0–2.7) ^b^	2.6 (2.2–3.1) ^a^	1.7 (1.1–3.2) ^b^	<0.001
LA:Ao	Median (min–max)	1.4 (1.2–1.9) ^c^	1.8 (1.5–3.0) ^b^	2.3 (1.6–4.2) ^a^	<0.001
LVDDn	Mean ± SD	1.38 ± 0.10 ^b^	1.93 ± 0.27 ^a^	2.09 ± 0.28 ^a^	<0.001
LVSDn	Mean ± SD	0.86 ± 0.12 ^c^	1.55 ± 0.28 ^a^	1.13 ± 0.26 ^b^	<0.001
FS (%)	Mean ± SD	34.9 ± 6.6 ^b^	13.4 ± 5.9 ^c^	41.5 ± 9.8 ^a^	<0.001
E Mitral (m/s)	Mean ± SD	0.67 ± 0.11 ^c^	1.05 ± 0.39 ^b^	1.36 ± 0.33 ^a^	<0.001

Data are expressed as mean ± standard deviation (SD) or median (range). Different superscript letters along rows mean significant different values (*p* ≤ 0.017); Abbreviations: N, number of dogs, DCM, dilated cardiomyopathy, MMVD, myxomatous mitral valve disease; ACVIM, American College of Veterinary Internal Medicine; CT, cardiac treatment; OT, other treatment, NT, no treatment; LA, left atrial diameter; Ao, aortic diameter; LA:Ao, left atrial diameter to aortic diameter ratio; LVDDn, left ventricular diastolic diameter normalized to body weight; LVSDn, left ventricular systolic diameter normalized to body weight; FS, fractional shortening; E mitral, mitral valve maximal E wave velocity.

**Table 3:** Results of Spearman’s rank correlation test showing the correlation between serum concentration of Gal-3 and clinical and echocardiographic variables.

Variable	r	*p*-Value
Age (years)	0.47	<0.001
BW (kg)	−0.45	<0.001
LA (cm)	−0.22	0.059
Ao (cm)	−0.38	0.001
LA:Ao	0.15	0.194
LVDDn	0.20	0.088
LVSDn	−0.19	0.101
FS (%)	0.43	<0.001
E Mitral (m/s)	0.11	0.369

Abbreviations: BW, bodyweight; LA, left atrial diameter; Ao, aortic diameter; LA:Ao, left atrial diameter to aortic diameter ratio; LVDDn, left ventricular diastolic diameter normalized to body weight; LVSDn, left ventricular systolic diameter normalized to body weight; FS, fractional shortening; E mitral, mitral valve maximal E wave velocity.

## Data Availability

The original contributions presented in the study are included in the article. Further inquiries can be directed to the corresponding author.
